# COUPLING OF WITHIN-PERSON CHANGES IN SLEEP QUALITY AND SUBJECTIVE COGNITION IN COMMUNITY-DWELLING ADULTS

**DOI:** 10.1093/geroni/igae098.3041

**Published:** 2024-12-31

**Authors:** Jose Diaz, Soomi Lee, Lynn Martire, Martin Sliwinski

**Affiliations:** The Pennsylvania State University, University Park, Pennsylvania, United States; The Pennsylvania State University, University Park, Pennsylvania, United States; The Pennsylvania State University, University Park, Pennsylvania, United States; Pennsylvania State University, University Park, Pennsylvania, United States

## Abstract

Subjective cognitive decline is not a mere complaint by older adults; it is predictive of later development of objective cognitive decline and dementia. Sleep plays a pivotal role in maintaining cognitive, physical, and mental health, especially in older adults. While studies document age-related declines in sleep quality, limited research explores whether within-person changes in sleep quality are associated with within-person changes in subjective cognition (“coupling effect”). In this study, we examined the dynamic coupling relationship between sleep quality and subjective cognition in older adults over multiple years, adjusting for potential influence of self-report bias (gauged by self-rated health). We used nine waves’ longitudinal data from the Transitions in Health and Relationships study (n=131, Mage=77.09). Multilevel models indicated that changes in sleep quality were significantly associated with changes in subjective cognitive function (β=0.044, 95% CI [0.004, 0.084], p=.031), signifying a coupling effect independent of baseline sleep quality, sociodemographic covariates, and self-rated health. Furthermore, changes in self-rated health moderated the association between changes in sleep quality and subjective cognition (β=-0.047, 95% CI [-0.088, -0.005], p=.028), such that increases in self-rated health weakened the effect of increases in sleep quality on subjective cognition. These findings underscore the significance of within-person changes in sleep quality in predicting subjective cognitive decline in older adults. Notably, while the association was independent of self-report bias, it was weaker for those who reported their overall health was improved. Future work should focus on maintaining or improving sleep quality in order to prevent or delay subjective cognitive decline.

